# Effect of Associated Autoimmune Diseases on Type 1 Diabetes Mellitus Incidence and Metabolic Control in Children and Adolescents

**DOI:** 10.1155/2016/6219730

**Published:** 2016-07-20

**Authors:** Aleksandra Krzewska, Iwona Ben-Skowronek

**Affiliations:** Department of Pediatric Endocrinology and Diabetology, Medical University of Lublin, 6 Professor A. Gebali Street, 20-093 Lublin, Poland

## Abstract

Type 1 diabetes mellitus (T1DM) is one of the most common chronic diseases developing in childhood. The incidence of the disease in children increases for unknown reasons at a rate from 3 to 5% every year worldwide. The background of T1DM is associated with the autoimmune process of pancreatic beta cell destruction, which leads to absolute insulin deficiency and organ damage. Complex interactions between environmental and genetic factors contribute to the development of T1DM in genetically predisposed patients. The T1DM-inducing autoimmune process can also affect other organs, resulting in development of additional autoimmune diseases in the patient, thereby impeding diabetes control. The most common T1DM comorbidities include autoimmune thyroid diseases, celiac disease, and autoimmune gastritis; additionally, diabetes can be a component of PAS (Polyglandular Autoimmune Syndrome). The aim of this review is to assess the prevalence of T1DM-associated autoimmune diseases in children and adolescents and their impact on the course of T1DM. We also present suggestions concerning screening tests.

## 1. Introduction

Diabetes is the most common chronic metabolic disease diagnosed in children and adolescents. Although it is not contagious, the disease is the first and only condition regarded by the United Nations as an epidemic of the 21st century [1].

In most parts of the world, type 1 diabetes is the most prevalent chronic disease in the population under 18 years of age although there are no reliable data available from many countries. There are significant differences in the incidence of the disease among different countries, with the lowest rates reported from China and Venezuela (0,1 per 100 000 people per year) and the highest in Finland and Sardinia (37 per 100 000 people per year) [2].

The results of international research (DIAMOND and EURODIAB) reveal an increasing trend in diabetes prevalence in most regions of the world, with the highest growth dynamics in the youngest age group [2].

The global increase in T1DM prevalence is a well-known fact; the incidence of type 1 diabetes in children worldwide has been growing at a rate from 3 to 5% per year since the 1960s, with the highest rate reported from fast developing countries [3–7].

The background of T1DM is probably associated with the autoimmune process of destruction of pancreatic beta cells by autoantibodies, which leads to absolute insulin deficiency and organ damage. However, there is no evidence that the destruction of the pancreatic beta cells is caused by the autoantibodies. The etiopathogenesis of this disease is complex and multifactorial. Most probably, the presence of many factors initiating or modulating the immune response leads to development of the disease [8].

As reported by literature, genetic factors have a crucial effect on the development of T1DM [9].

Genetic predisposition is related to genes located in the major histocompatibility complex (MHC) on chromosome 6p21.3, accounting for at least 40% of the family history of the disease. Depending on the age of the disease onset, between 30% and nearly 50% of individuals with type 1 diabetes have a specific heterozygous genotype comprising alleles HLA-DQA1^*∗*^0501DQB1^*∗*^0201 (referred to as DQ2)/DQA1^*∗*^0301DQB1^*∗*^0302 (referred to as DQ8) with associated alleles DR3 and DR4 (referred to as the DR3/DR4 genotype). HLA antigens with their protective effect, such as HLA-DQA1^*∗*^0102DQB1^*∗*^0602 (referred to as DQ6) in combination with DR2 alleles, should also be mentioned. Predisposition to type 1 diabetes is associated with a polymorphism or mutation of many other genes. The most important of these include the insulin gene promoter (INS-VNTR, insulin-linked variable number of tandem repeats), a gene encoding a T-lymphocyte activation receptor (CTLA-4, cytotoxic T-Lymphocyte Antigen 4), and the protein tyrosine phosphatase gene N22 (PTPN22, protein tyrosine phosphatase, nonreceptor type 22) [10].

However, environmental (quantitative or qualitative) factors seem to have a major effect on the growing T1DM incidence observed over the last decade, as it is unlikely that genetic factors can operate within such a short time [11, 12].

More frequently, T1DM is diagnosed in patients with low genetic predisposition. Therefore, environmental factors seem to be the major trigger initiating the disease by stimulation of immune response against beta cells or overloading already affected beta cells, thereby accelerating the disease process, or by mitigation of the protective effect of environmental factors [2, 3].

Despite the decades of research, environmental factors promoting the development of pancreatic islet autoimmunity have not been elucidated yet [10]. Gut microbiota is one of the important environmental factors in development of T1DM. It may be involved in the progression from *β*-cell autoimmunity to clinical disease rather than in the initiation of the disease process [11].

Some publications emphasize the impact of viral infections or vitamin D3 deficiency on the development of autoimmunity in pancreatic beta cells. As documented in a TRIGR study, breast-feeding or, if this is not possible, administration of casein hydrolysates reduces the risk of development of pancreatic beta-cell autoimmunity in genetically predisposed children [13].

Currently, it is believed that there is a subclinical disease period (prediabetes) before the onset of clinical symptoms prior to destruction of approx. 90% of pancreatic beta cells. It is associated with the onset of autoimmune response and appearance of autoantibodies, sometimes many years before the manifestation of the disease [14].

In recent decades, significant progress has been achieved in clarification of the involvement of autoantigens in autoimmune processes. A number of receptors, enzymes, and hormones have been identified as target antigens in organ-specific autoimmune diseases [15].

“Islet autoantibodies” is a general term for any group of antibodies directed against Langerhans islets or, in some circumstances, against insulin-producing beta cell autoantigens. The list of autoantibodies and autoantigens that have been discovered is long (see “*Selected Autoantibodies in T1DM*” below), but in most cases they are difficult to determine and/or they are not sufficiently sensitive and specific to justify application thereof in everyday clinical practice [16]. In children and adolescents, the following autoantibodies are detected at diagnosis of the disease: anti-glutamic acid decarboxylase (anti-GAD), anti-islet cell (ICA), anti-insulin (IAA), anti-tyrosine phosphatase (IA2), and anti-zinc transporter 8 (ZnT8) antibodies. David Leslie, Peter Lipsky, and Abner Notkins emphasize the key role of these antibodies in the qualification of the disease, assessment of its activity, and the risk of progression and in prediction of the symptomatic phase of the disease [17, 18].

Selected autoantibodies in T1DM are as follows, adapted from [16]:

Insulin, insulin processing, and insulin Storage:

 Carboxypeptidase H autoantibodies.
 Insulin autoantibodies (IAA).
 Proinsulin autoantibodies.
 Zinc transporter 8 protein (ZnT8A).

Protein tyrosine phosphatases:

 Insulinoma 2- (IA-2-) associated autoantibodies (IA-2A).
 IA-2*β* autoantibodies (IA-2*β*A).

Enzymes:

 Carbonic anhydrase II.
 Chymotrypsinogen-related 30 kDa pancreatic autoantibody.
 DNA topoisomerase II autoantibodies.
 Glutamic acid decarboxylase (GAD) autoantibodies (GADA).
 51 kDa aromatic-L-amino-acid decarboxylase autoantibodies.

Miscellaneous:

 Aminoacyl-tRNA synthetase autoantibodies.
 Glima 38 autoantibodies.
 GLUT2 autoantibodies.
 Glycolipid autoantibodies.
 GM2-1 islet ganglioside autoantibodies.
 Heat shock protein autoantibodies.
 Islet cell surface autoantibodies (ICSA).
 Islet cell-specific 38 kDa autoantibodies.
 Islet-cell cytoplasmic autoantibodies (ICA).
 52 kDa RIN (rat insulinoma) autoantibodies.

It has been evidenced that the development of type 1 diabetes increases the risk of other autoimmune diseases. This is related to genetic susceptibility to development of these diseases. The autoimmune process progressing in pancreatic beta cells can also affect other organs, resulting in development of organ-specific autoimmune diseases, or various nonspecific tissues and organs, leading to development of organ-nonspecific autoimmune diseases [19].

The most frequent comorbidities of type 1 diabetes include Hashimoto's thyroiditis and Graves' disease collectively referred to as autoimmune thyroid diseases (15–30%), celiac disease (4–9%), autoimmune gastritis/pernicious anemia (5–10%), Addison's disease (0,5%), and vitiligo (2–10%). The frequency of occurrence of these diseases is increased in children and adolescents with T1DM in comparison to healthy children [20, 21]. These diseases are associated with the presence of specific autoantibodies in blood serum, which can be detected prior to the development of clinically overt disease [20–22]. There are ongoing investigations of the genetic basis of the coincidence of T1DM and other autoimmune diseases. The results are described in Table 1 [23]. The occurrence of other autoimmune diseases in patients with type 1 diabetes deteriorates the quality of life and increases morbidity and mortality [22, 23].

The aim of this paper is to review the literature on autoimmune diseases associated with type 1 diabetes in children and adolescents.

## 2. Autoimmune Type 1 T1DM Comorbidities

### 2.1. T1DM and Autoimmune Thyroid Diseases (AITD)

Hashimoto's thyroiditis and Graves' disease, referred to as autoimmune thyroid diseases (AITD), are the most prevalent autoimmune diseases in children and adolescents with type 1 diabetes [26].

Their incidence is 2–4-fold higher than in the general population [27], with Hashimoto's thyroiditis as the most common clinical form (14–28%) [21] and Graves' disease diagnosed less frequently (0,5–7%) [21, 28, 29].

AITD is characterized by lymphocytic infiltration caused by loss of immunological tolerance to thyroid autoantigens, which is manifested in production of autoantibodies. Consequently, thyroid exhibits various degrees of function impairment. The autoantibodies are directed against specific thyroid proteins, thyroglobulin (ATG), thyroxin peroxidase (ATPO), and TSH receptor (A-TSHR) [30]. The presence of thyroid antibodies ranges from 2,9% to 4,6% in the general population and from 12,1% to 23,4% in children and adolescents with T1DM; the antibodies are detected in only 17–25% of patients with T1DM at the time of diagnosis. In a majority of cases, they appear within 2,5–3 years of the progression of the disease [19]. AITD very rarely precedes the diagnosis of T1DM [19, 26, 27]. The presence of anti-thyroid antibodies in T1DM patients increases with age, duration of the disease, and long-term persistence of anti-GAD antibodies [27]. Glutamic acid dehydrogenase, against which anti-GAD antibodies are produced, induces conversion of glutamic acid to gamma-aminobutyric acid (GABA). In the thyroid, GABA is located in follicular cells and is involved in regulation of thyroid hormone secretion. Probably, autoimmune response expands with time and covers not only beta cells but also thyrocytes. A strong correlation has been observed between the presence of thyroid autoantibodies and the female sex [28]. In animal models, likewise in T1DM patients, estradiol accelerates progression of autoimmune diseases by disturbance of the action of type 2 T-helper lymphocytes (Th2), whereas androgens exert a protective effect. The fact that the adolescence period is associated with the higher incidence of autoantibodies confirms the effect of female sex hormones on the development and progression of the autoimmune process [19]. Despite the presence of anti-thyroid antibodies in blood serum, a majority of patients do not present with symptoms of thyroid dysfunction [19].

AITD more often produces signs of hypothyroidism (Hashimoto's thyroiditis) and less frequently of hyperthyroidism (Graves' disease or the hyperactive phase of Hashimoto's thyroiditis).

In patients with T1DM, the presence of haplotypes HLA-DQA1^*∗*^0301, DQB^*∗*^0301, and DQB1^*∗*^0201 is associated with development of hyperthyroidism, whereas haplotype DQA1^*∗*^0501 is related to hypothyroidism. Haplotype HLA-DQB1^*∗*^05 seems to have a protective role in the development of AITD [27, 31]. As shown by literature data, as many as 30% of patients with type 1 diabetes can be affected by hypothyroidism [21]. Overt hypothyroidism is diagnosed in 4–18% of T1DM patients [19], which is a higher level than that in the general population (5–10%) [21]; in turn, subclinical hypothyroidism is diagnosed in 40–55% of patients [19]. Treatment of hypothyroidism involves replacement therapy based on levothyroxine administration. Subclinical hypothyroidism in patients with T1DM, in particular those with the values of TSH ≥ 10 *μ*lU/L, may be associated with hypoglycemia and growth retardation [27, 32, 33].

Physiological effects of the action of thyroid hormones include enhanced intestinal glucose absorption, glycogenolysis, and insulin catabolism in the liver. These mechanisms have a hyperglycemic effect, and even slight changes in the levels of thyroid hormones can increase the risk of hypoglycemia [33]. Hypothyroidism in children with type 1 diabetes is frequently associated with hypoglycemia resulting from increased insulin sensitivity. Growth disorders diagnosed in these children are associated with chronic hypoglycemia and thyroid hormone deficiency [34].

On the other hand, no effect of subclinical hypothyroidism on the growth, BMI, and glycemia control in T1DM patients has been reported by other researchers [26, 27].

Nevertheless, subclinical hypothyroidism in patients with T1DM may be associated with an adverse lipid profile; therefore, early treatment reduces the risk of development of hyperlipidemia and atherosclerotic cardiovascular diseases [35].

Hyperthyroidism in Graves' disease or in Hashimoto's hyperactive phase (Hashitoxicosis) is less frequent than hypothyroidism in T1DM patients; yet, its incidence is higher than in the general population. Investigations have shown a reduced level of TSH and increased levels of T4 and T3, as well as the presence of anti-TSH receptor antibodies in Graves' disease. Dost et al. have demonstrated that hyperthyroidism in children with T1DM is associated primarily with acute diabetes complications, for example, ketoacidosis or hypoglycemia, and arterial hypertension. However, no effect on long-term metabolic control and insulin demand has been reported [36]. In contrast, other studies indicate that hyperthyroidism leads to increased glucose demand resulting from increased metabolism of the entire organism [37]. This, in turn, leads to stimulation of gluconeogenesis and glycogenolysis, decreased insulin sensitivity, increasing glucose uptake in muscle, and lipolysis, leading to deterioration of metabolic control [38]. Hyperthyroidism is treated with thyrostatic drugs (such as methimazole), radioiodine, and propranolol. In accordance with the guidelines of the American Diabetes Association (ADA) and the International Society for Pediatric and Adolescent Diabetes (ISPAD), assessment of thyroid function and thyroid autoantibodies in children with T1DM should be performed at the time of diabetes diagnosis. If the results are normal, the examinations should be repeated every two years or more frequently if AITD symptoms occur [39, 40].

As already mentioned, no antithyroid antibodies are usually detected at the time of diagnosis of T1DM; they appear later in the course of the disease [19]. For this reason, if no antibodies are present, serological screening and evaluation of thyroid function should be performed every 12 months or 6 months in the case of the presence of autoantibodies (ATG, ATPO) [20]. Furthermore, the thyroid should be examined with ultrasound at least once a year if autoantibodies are detected or a goiter is diagnosed [39, 40].

### 2.2. Type 1 T1DM and Celiac Disease

Celiac disease (gluten-dependent celiac disease) is a chronic autoimmune enteropathy occurring in genetically predisposed individuals and caused by intolerance to gluten, that is, a mixture of plant proteins contained in certain European cereals (wheat, rye, and barley). Gluten induces a specific immune reaction and production of autoantibodies (antireticulin (ARA), antiendomysial (EmA), antigliadin (AGA), and anti-tissue transglutaminase (ATG)) as well as atrophy of intestinal villi [41, 42]. The following types of the disease have been distinguished: silent, potential, latent, and classical [41]. Celiac disease (CD) is diagnosed in 1–5,5% of the general pediatric population, with the lowest incidence in Japan and the highest rates in the Sahrawi population [41].

In children with T1DM, the CD incidence is higher and ranges from 0,6 to 16,4% [43]. The estimates demonstrate a 20-fold higher prevalence of celiac disease in children with diabetes than that in the general population [44]. In approx. 90% of cases, celiac disease develops in the course of type 1 diabetes and it is diagnosed by screening tests. Celiac disease rarely occurs before the diagnosis of diabetes [42, 45]. Factors predisposing to celiac disease in patients with T1DM include the female sex and diabetes diagnosed at an early age [43, 46].

Most probably, there is a multifactorial relationship between both diseases. A key genetic factor in both cases is the presence of genotypes HLA-DQ8 (DQB1^*∗*^0302-DQA1^*∗*^0301) and DQ2 (DQB1^*∗*^0201-DQA1^*∗*^0501) [47]. It has also been suggested that wheat gluten is an environmental factor that induces development of T1DM [13].

The diagnosis of celiac disease is based on determination of specific antibodies and confirmation of the disease by a biopsy of the small intestine. Antiendomysial (EMA) and anti-tissue transglutaminase (ATG) antibodies are the two most important antibodies in the diagnosis of celiac disease. Currently, determination of antigliadin antibodies is not recommended in the diagnostics of CD, as they are less sensitive and less specific than EMA and TTG [21].

Due to the higher incidence of IgA deficiency in patients with celiac disease than in the general population, the levels of total IgA should be determined and, if these are low, IgG antibodies should be assessed [48].

Determination of antibodies present in celiac disease is crucial, since most cases are asymptomatic. In children with T1DM, celiac disease is most often diagnosed in the silent or potential phase, prior to the onset of clinical symptoms of the disease [49, 50]. Intestinal villus atrophy leads to malabsorption of nutrients, fat-soluble vitamins, B vitamins, folic acid, iron, or calcium. Therefore, celiac disease in children with type 1 diabetes may be associated with hypoglycemia episodes, growth impairment, body weight loss, osteopenia, osteoporosis, or anemia. Chronic malabsorption of vitamin D and calcium can result in bone demineralization, osteoporosis, and rickets. Osteopenia may be enhanced by the reduction of the concentration of IGF-1 and sex steroids in children with delayed puberty. Delayed bone maturation and growth disorders are often accompanied by disturbances in sexual maturation. Furthermore, women can also be affected by reproductive disorders, for example, infertility or recurrent miscarriages. Patients with celiac disease may also present with various neurological complications such as epilepsy with occipital calcifications, cerebellar ataxia, or cerebral vasculitis. Neurological disorders seem to be comorbidities rather than diseases caused by gluten intolerance [49].

The treatment of celiac disease involves introduction of a strict, lifelong gluten-free diet [41]. This results in clinical improvement in patients with T1DM [51] and is recommended for patients with classical or subclinical symptoms of the disease [49].

Recent studies have revealed that the gluten-free diet in patients with type 1 diabetes has drawbacks as well. For instance, the cooperation in compliance with the diet is poorer in patients affected by both diseases. In turn, the role of the diet in optimization of metabolic control is debatable. Gluten-free diet can result in increased weight and BMI in patients with T1DM as a consequence of consumption of great amounts of saturated fatty acids in foods with a high glycemic index and decreased amounts of protein and fiber. This, in turn, can contribute to development of microvascular complications and impair metabolic control. For all these reasons, it is essential that gluten-free diet should be introduced with care and under supervision of experienced specialists [19, 52].

On the other hand, poor compliance with gluten-free diet in patients with celiac disease may lead to unexplained hypoglycemia episodes [52] or development of malignancies (mainly lymphoma) in adult life [53].

Guidelines issued by various diabetic societies do not provide uniform recommendations for CD screening in patients with T1DM.

In the 2014 guidelines, the American Diabetes Association (ADA) recommends determination of ATG or EMA of the IgA class shortly after diagnosis of diabetes and exclusion of IgA deficiency. Screening should be carried out in children with a family history of CD, presenting with atypical or typical symptoms of celiac disease (stunted growth, absence of weight gain, weight loss, diarrhea, tympanites, and abdominal pain), symptoms of malabsorption and frequent unexplained episodes of hypoglycemia, or deteriorated glycemia control [54].

The International Society for Pediatric and Adolescent Diabetes (ISPAD) recommends determination of TTG and EMA of the IgA class at the time of diagnosis of the disease and annual determination over the first five years of the disease followed by biennial tests. The determinations should be performed more frequently in the presence of clinical symptoms suggesting CD or occurrence of CD in first-degree relatives [39]. All recommendations suggest that children with a recurrently positive antibody titer should be referred to a pediatric gastroenterologist for a biopsy and, when CD is confirmed, a gluten-free diet should be introduced and supervised by an experienced dietician.

In turn, according to the recommendations of the European Society for Pediatric Gastroenterology, Hepatology, and Nutrition (ESPEGHAN), an intestinal biopsy may be waived if the diagnosis of CD is based on typical symptoms, presence of specific autoantibodies, a particularly high level of TTG IgA (10-fold higher than the upper limit), and a specific genotype (HLA-DQ2/DQ8). The decline in the antibody titer and the clinical response to gluten-free diet confirm the diagnosis. Gluten provocation tests and repeated biopsies are necessary only in some patients with an uncertain diagnosis or a high probability of disease in the absence of antibodies. Moreover, in groups with an increased risk of celiac disease, diagnostics can start with a genetic test, if this is possible. In the case of a negative result, celiac disease can virtually be excluded and diagnostics can be terminated at this stage. In turn, when a positive result is obtained, determination of TTG antibodies is recommended. However, due to its cost, the determination is not carried out routinely in clinical practice [55].

### 2.3. T1DM and Autoimmune Gastritis

Autoimmune gastritis is an asymptomatic and common disease with higher prevalence among children with T1DM than in the general population. Clinical symptoms appear as a consequence of progression of the disease and development of atrophic gastritis with iron and vitamin B12 deficiency (pernicious anemia). Atrophic gastritis may be a cause of carcinoid tumors and gastric cancer. Laboratory diagnosis of autoimmune gastritis is based on determination of serum biomarkers, for example, anti-parietal cell autoantibodies (APCA) and antibodies directed against an inner factor, and in the case of atrophic gastritis, on determination of the concentration of pepsinogen and gastrin [56, 57]. In patients with T1DM, the APCA prevalence rate is 3–30% (5,3–7,2% in children and adolescents) [58–60], while among healthy children aged 0–15, the rate is substantially lower, that is, 0,3–1,98% [58, 60, 61]. The presence of a specific HLA haplotype (HLA-DQA1^*∗*^0501-DQB1^*∗*^0301) in patients with T1DM increases the risk of autoimmune gastritis. This genotype is also associated with the presence of GAD and ATPO antibodies [58, 59].

Literature reports controversies concerning the impact of other factors, for example, age, sex, or duration of the disease, on the presence of APCA. The presence of APCA is associated with an older age of T1DM patients, longer duration of the disease [62], and the female sex [19]. In contrast, other studies have demonstrated no relationship between the presence of APCA and these factors [58, 59]. A correlation has been found between the presence of APCA, anti-thyroid antibodies, and anti-GAD antibodies in patients with T1DM [59]. This correlation has been noted both in the adult population and in children with T1DM. An explanation for the increased incidence of autoimmune diseases in patients with type 1 diabetes may be the common etiological factor, that is, the genetic background, in this case probably related to chromosome 4. Another possible explanation is the hypothesis that the pancreas, thyroid, and stomach present common autoantigens [58]. The GAD antigen is present in the pancreas as well as gastric submucosa and mucosa, where it is involved in conversion of glutamic acid to gamma-aminobutyric acid (GABA), promoting production of hydrochloric acid by parietal cells. Probably, the T-cell response to the GAD antigen begins in the pancreas; next, the autoimmune process proceeds and active T-cells initiate destruction of other neuroendocrine tissues containing the same or similar enzymes [63].

Damage to gastric parietal cells by APCA leads to autoimmune gastritis characterized by hypo- or achlorhydria, hypergastrinemia, and a reduced level of pepsinogen I. Chronic hypergastrinemia induces hyperplasia in the parietal mucosa of enterochromaphilic cells (ECL), which can cause dysplasia (atrophic gastritis) and gastric carcinoid tumors [57].

Patients with T1DM and serum APCA are at a greater risk of gastric autoimmunity, anemia with iron deficiency, and/or pernicious anemia. Yet, overt autoimmune gastritis is rare in children with T1DM [60].

Periodic examinations detecting autoimmune gastritis are particularly crucial in T1DM patients with serum GAD and/or anti-thyroid antibodies, as they help prevent and introduce early treatment of iron and vitamin B12 deficiencies as well as precancerous and cancerous lesions [57].

Unfortunately, there are no guidelines for management of patients with diagnosed APCA. Since autoimmune gastritis is usually asymptomatic, annual determinations of blood morphology, ferritin, vitamin B12, and gastrin levels are recommended in patients with APCA [19, 57]. In patients with a high APCA titer and hypergastrinemia, gastroscopy with a multiple biopsy should be performed [63]. At diagnosis, children with T1DM should have APCA levels determined. In the case of a negative result, the examination should be repeated every 3 years. In the case of clinical indications, the determinations must be performed immediately, since autoimmune gastritis can develop at any age. In patients with autoimmune thyroid disease and/or GAD antibodies persisting for over 5 years, APCA should be determined every 2 years, due to the increasing risk of development of gastric autoimmunity [19, 57, 58, 63].

### 2.4. T1DM and Adrenal Autoimmunity

In developed countries, approx. 80% of primary adrenal insufficiency, Addison's disease (AD), is caused by an autoimmune process [64].

Adrenal damage leads to impaired production of glucocorticosteroids, mineralocorticoids, and androgens accompanied by high serum levels of ACTH and high plasma renin activity [65].

It rarely occurs in the general population, with an incidence rate from 90 to 140 people per million. Over half of patients with AD present with other autoimmune diseases, for example, as components of Polyglandular Autoimmune Syndromes (PAS) [64, 66]. The presence of antibodies against the adrenal cortex (adrenal cell antibody, ACA) is characteristic for the autoimmune adrenal insufficiency [67]. A main autoantigen is the microsomal cytochrome P450 enzyme 21-hydroxylase, which converts alpha progesterone and progesterone to 11-deoxycortisol and 11-deoxycorticosterone [19, 68]. T1DM in children and adolescents usually precedes the development of AD [66], the incidence rate of which is approx. 1-2% in adult T1DM patients. In turn, the incidence of T1DM in AD patients is 10–18% [19]. Most frequently, type 1 diabetes can develop in patients with PAS-1 and PAS-2.

The incidence of ACA in adult T1DM patients is 0–4% [66, 69, 70]. The value increases when type 1 diabetes is accompanied by autoimmune thyroid disease. It has been found that approx. 70% of T1DM patients and ACA autoantibodies present with AITD as well [69]. T1DM and AD have a common genetic background mainly associated with the HLA class II system (haplotype DRB1^*∗*^0404/DQ8 and DRB1^*∗*^0301/DQ2) [69]. There are contradictory reports concerning factors that determine the development of ACA in patients with T1DM. Some investigations have shown a correlation between the female sex and long duration of diabetes, while other researchers have found no relationship between ACA and these parameters or the age at which T1DM is diagnosed [19, 61, 66, 68, 69].

AD can be suspected based on typical clinical symptoms of adrenal insufficiency, for example, fatigue (due to orthostatic hypotension and hypoglycemia), weight loss, decreased appetite, nausea, desire to consume salty foods, and discoloration of the skin and mucous membranes. Moreover, unexplained hypoglycemic episodes may occur and insulin demand may be decreased in patients with T1DM [68]. Hypoglycemia is not a common symptom of Addison's disease in patients with type 1 diabetes and in those without diabetes. Nevertheless, hypoglycemia episodes are associated with glucocorticosteroid deficiency resulting in enhanced insulin sensitivity of target tissues, increased glycolysis, and reduced glycogenesis and gluconeogenesis [66]. Recurrent unexplained hypoglycemia episodes should prompt the doctor potential Addison's disease in T1DM patients [71]. AD treatment is based on chronic substitution of adrenal cortex hormones, glucocorticoids and mineralocorticoids, and, after adolescence, administration of androgens [67].

Currently, there are no guidelines available for management of patients with diabetes and possible adrenal autoimmunity. Routine determination of ACA is not recommended; hence the risk and period of adrenal autoimmunity development in these patients are not predictable [19]. As suggested by literature, ACA determination is recommended in T1DM patients over 18 years of age only in the case of presence of clinical adrenal autoimmunity symptoms or every 5 years if the disease afflicts first-degree relatives [19, 23, 72].

### 2.5. T1DM and Vitiligo

Another autoimmune disorder associated with T1DM is vitiligo. The disease is characterized by loss of epidermal melanocytes leading to appearance of delimited discoloration of the skin. The etiopathogenesis of the disease is not clear. Probably, the development of vitiligo is influenced by both genetic and environmental factors. Three major hypotheses concerning the pathogenesis of vitiligo have been proposed. The “neural” hypothesis suggests that accumulation of toxic neurochemical substances released from the nerve endings damages melanocytes, which results in reduced melanin production. According to the “biochemical” hypothesis, the self-destruction of pigment cells is a result of accumulation of intermediate toxic products of melanin synthesis, impairment of defense against free radicals, and accumulation of excessive amounts of hydrogen peroxide. The third hypothesis emphasizes the important role of autoimmunity [73, 74]. Vitiligo often occurs with other autoimmune diseases [20, 21], and a majority of patients present with reactive T-cells in blood serum anti-melanocyte autoantibodies [73]. Anti-melanocyte antibodies are present in a substantial proportion of patients with vitiligo [74]. Additionally, a positive correlation has been observed between the presence of antibodies and the severity of the disease (increased incidence in patients with larger areas of affected skin). Vitiligo is mediated by Th1 response [75]. The destruction of melanocytes may be mediated by cytotoxic CD8 T-cells. A T-cell product of IL-2R and an increased CD8 : CD4 ratio is observed in persons with vitiligo. Perilesional HLA-DR production (MHC class II receptor) was reported in all patients with vitiligo, especially along suprabasal and basal keratinocytes, due to local T-cell reactivity. In addition, macrophages were more numerous in vitiligo in comparison to healthy people [76].

The incidence of vitiligo in the general population is 0,5%–1% [77], and over 50% of cases are diagnosed before the age of 20 [73]. Vitiligo may precede the development of T1DM [78]. Diabetes can develop in ca. 16–20% of vitiligo patients. Polyglandular autoimmune syndrome PAS-2 often starts with development of vitiligo [22], which is more frequent in patients with T1DM [20]. A common genetic background related to deficiency in the ATXN2 gene, which mediates abnormal metabolism of sugars and fats, has been suggested [79]. Moreover, vitiligo in patients with T1DM increases the risk of other diseases, particularly AITD and gastritis, which should be actively diagnosed [20, 23]. Unfortunately, there is no effective treatment of vitiligo [80]. The many methods of treatment include topical corticosteroids, vitamin-D derivatives, calcineurin inhibitors, photochemotherapy (psoralen plus UV-A (PUVA), psoralen with sunlight (PUVAsol)), phototherapy (UV-A, narrowband UV-B), and surgical techniques [81–86].

### 2.6. Polyglandular Autoimmune Syndromes (PAS)

Diabetes can be a component of Polyglandular Autoimmune Syndromes (PAS), a heterogeneous group of rare diseases defined as functional disorders in at least two of the endocrine glands and, possibly, in other organs. They occur in genetically predisposed individuals after activation of a factor that triggers abnormal immune response. Individual endocrinopathies may develop at different times and their symptoms are preceded by the presence of specific autoantibodies in serum. The first classification was proposed by Neufeld and Blizzard in 1980 [87]. The current classification distinguishes three types of PAS based on the clinical picture, genetic background, and the time of the onset of the disease (Table 2) [88].

Type 1 PAS, referred to as Blizzard's syndrome in literature, usually develops in 3–5-year-old children or younger adolescents. The diagnosis of PAS-1 requires the presence of at least two of the following conditions: Addison's disease, hypoparathyroidism, mucocutaneous candidiasis, and an evidenced AIRE gene mutation [22]. Other diseases constituting the syndrome include autoimmune hepatitis, primary hypothyroidism, vitiligo, pernicious anemia, and type 1 T1DM. PAS-1 is associated with a mutation of a single AIRE gene, which is located on chromosome 21, is characterized by autosomal recessive inheritance, and is involved in immune regulation. The disease typically begins in childhood and manifests itself by mucocutaneous fungal infection [22, 89]. Screening for anti-islet, anti-21-hydroxylase, anti-parietal cell (APCA), and anti-tissue transglutaminase antibodies as well as vitamin B12 deficiency is recommended in patients with PAS-1 [19].

Schmidt's syndrome (type 2 PAS) is the most prevalent type of PAS afflicting adults. The elements of this syndrome include Addison's disease, IgA deficiency, autoimmune thyroid diseases, primary hypothyroidism, hypogonadism, hypopituitarism, type 1 diabetes, Parkinson's disease, myasthenia gravis, celiac disease, vitiligo, alopecia, pernicious anemia, stiff person syndrome, malabsorption, hepatitis, and asplenia [22]. PAS-2 can frequently be diagnosed among family members of many generations. The first symptoms of the disease can be evident in the first decade of life, but its incidence peak is noted at the age of 25–30. The syndrome exhibits 3-fold higher frequency in women [24, 89]. Patients with PAS-2 should be periodically evaluated for diabetes (IAA, IA2, and GADA), thyroid diseases (e.g., A-TG, A-TPO), Addison's disease (21-OH hydroxylase), celiac disease (a-tTG), and autoimmune hepatitis (cytochrome P-450 enzymes) [19].

PAS-3 comprises the same spectrum of endocrine gland diseases as PAS-2, though without adrenal insufficiency. Three subtypes have been distinguished: (1) Hashimoto's disease with type 1 diabetes; (2) Hashimoto's disease with pernicious anemia; and (3) Hashimoto's disease with vitiligo and/or alopecia and/or other organ-specific and organ-nonspecific autoimmune diseases (celiac disease, hypogonadism, myasthenia gravis, sarcoidosis, rheumatoid arthritis, and Sjögren's syndrome). Similar to PAS-2, PAS-3 is characterized by polygenic inheritance and is associated with the HLA class II System [88, 89].

In literature, PAS-4 is distinguished when comorbid polyendocrinopathies cannot be classified as PAS-1, PAS-2, or PAS-3 [24, 87, 90].

The incidence of type 1 diabetes in the syndromes is 4–18% in PAS-1, 60% in PAS-2, and 14,5% in PAS-3. Although clinical manifestations of the disease are usually observed in the third decade of life, the first symptoms may appear in childhood. Therefore, every pediatrician should know PAS symptoms, especially in relation to multiple autoimmune comorbidities accompanying type 1 diabetes [19, 89, 91, 92].

### 2.7. T1DM and Organ-Nonspecific Autoimmune Diseases

In patients with type 1 diabetes,organ-nonspecific autoimmune diseases can develop, for example, juvenile idiopathic arthritis (JIA), Sjögren's syndrome, or psoriasis [89, 93] There are few investigations concerning these T1DM comorbidities, in particular in pediatric patients [89].

JIA is the most common organ nonspecific autoimmune disease in children. Diabetes onset occurs earlier in children with JIA: a median of 7.2 years versus 8.3 years in children without arthritis. Also, 88% of children with arthritis developed diabetes first, with the age median of diabetes onset of 5 years earlier than the age of arthritis diagnosis. A multicenter German and Austrian study involving 54 911 patients under 16 years of age with type 1 diabetes demonstrated a 0,19% incidence of JIA in these patients, which was a significantly higher value than that in the general population (0,05%). The disease was shown to affect girls twice as often as boys [93].

The T1DM and JIA comorbidity has increased in recent decades [93, 94].

Furthermore, the coexistence of T1DM and JIA increases the risk of development of AITD and CD [93]. Literature provides growing evidence for the common genetic background of JIA and T1DM, which is associated with a mutation in the PTPN22 gene encoding an enzyme inhibiting the T-cell activation pathway [95, 96]. A majority of JIA cases seem to precede development of T1DM [89, 97], which suggests that T1DM is a consequence of steroid therapy in patients with JIA. Steroid therapy impairs glycemic control in patients with T1DM and JIA [89]. There are also data concerning the development of T1DM upon administration of etanercept [98]. There is a need for further population research on these comorbidities and the effect of steroid and biological JIA therapy on metabolic control in patients with type 1 diabetes. Nevertheless, the significantly higher incidence of JIA in T1DM patients should prompt doctors to monitor patients with type 1 diabetes carefully. Steroid therapy substantially increases the insulin demand in T1DM patients.

Psoriasis is a chronic inflammatory disease of the skin, nails, and joints with an immune background, affecting 2.0–3.5% of the world population [99]. In a meta-analysis carried out by Cheng et al., it was found that the risk of diabetes in psoriasis patients was 1.42-fold higher than in healthy individuals [100]. There is increasing evidence suggesting that children with psoriasis exhibit a higher incidence of such comorbidities as diabetes, obesity, rheumatoid arthritis, Crohn's disease, or mental disorders, which should be diagnosed early and treated appropriately [99, 101].

Sjögren's syndrome is a systemic autoimmune disease mostly affecting lacrimal and salivary glands. The spectrum of the disease ranges from dryness syndrome to systemic disease of exocrine glands [102]. Literature provides single reports of the development of diabetes in Sjögren's syndrome [103]. Organ nonspecific autoimmune diseases are usually regarded as components of Polyglandular Autoimmune Syndromes [25, 89].

The impact of T1DM-associated autoimmune diseases on the course of T1DM and suggestion concerning screening tests are shown in Table 3.

## 3. Conclusions

The conclusions of this paper are as follows:

1. T1DM enhances the risk of development of other autoimmune diseases in children and adolescents.
2. Coexistence of type 1 diabetes and other autoimmune diseases impairs glucose metabolism, impedes effective insulin therapy, and deteriorates diabetes control.
3. Monitoring of autoimmune responses in patients with autoimmune diseases is crucial, as these responses may affect other organs.

## Figures and Tables

**Table 1:** Analysis of T1DM loci on the basis of genome-wide linkage analyses and loci for occurrence of other autoimmune diseases in the same region [23].

Locus	Other autoimmune disease
IDDM1	All autoimmune diseases
IDDM3	Celiac disease
IDDM5	Rheumatoid arthritis
IDDM6	Rheumatoid arthritis, AITD, SLE
IDDM8	Rheumatoid arthritis
IDDM9	Rheumatoid arthritis
IDDM12 (CTLA4)	Rheumatoid arthritis, multiple sclerosis, AITD, Addison disease
IDDM13	Rheumatoid arthritis
16q22–q24	Psoriasis, asthma, celiac disease
DXS998	Rheumatoid arthritis

**Table 2:** Characteristics of PAS, adapted from Betterle et al. [24, 25].

PAS	Major components and autoantigen targets		Minor components and autoantigen targets		Genetic
I	Addison's disease	21-Hydroxylase	Gonadal failure	P450 side-chain cleavage enzyme, 17a-hydroxylase	AIRE gene mutations (chromosome 21)
	Chronic hypoparathyroidism Chronic candidiasis (at least 2 present)	Calcium-sensing receptor	Vitiligo	SOX9, SOX10, tyrosinase	
			Alopecia	Tyrosine hydroxylase	
II	Addison's disease (always present)	21-Hydroxylase	Atrophic gastritis	H+/K+ pump ATPase	Polygenic inheritance (HLA-DR3, chromosome 6)
			Pernicious anaemia	Intrinsic factor	
	Thyroid autoimmune diseases	TSH-receptor Thyroid peroxidase Thyroglobulin	Celiac disease	Transglutaminase	
			Autoimmune hepatitis	P450 (IID6, IA2)	
	Type 1 diabetes mellitus	Glutamic acid decarboxylase Tyrosine-phosphatase Insulin	Hypophysitis and so forth	68, 49, 43 kD from human	
III	Thyroid autoimmune diseases associated with endocrinopathy other than Addison's disease	TSH-receptor Thyroid peroxidase Thyroglobulin	Celiac disease	Transglutaminase	Polygenic inheritance (HLA-DR3, chromosome 6)
			Autoimmune hepatitis	P450 (IID6, IA2)	
			Myasthenia gravis and so forth	Acetylcholine receptor	
IIIA	Autoimmune thyroiditis	Thyroid peroxidase			
	Type 1 diabetes mellitus	Glutamic acid decarboxylase Tyrosine-phosphatase Insulin			
IIIB	Autoimmune thyroiditis	Thyroid peroxidase			
	Pernicious anaemia	Intrinsic factor			
IIIC	Autoimmune thyroiditis	Thyroid peroxidase			
	Vitiligo	SOX9, SOX10, tyrosinase			
	Alopecia	Tyrosine hydroxylase			
	Other organ-specific autoimmune disease				
IV	Combination of organ-specific autoimmune diseases that do not fall in the previous types		Gonadal failure	P450 side-chain cleavage enzyme, 17a-hydroxylase	Polygenic inheritance (HLA-DR3, chromosome 6)
			Vitiligo	SOX9, SOX10, tyrosinase	
			Alopecia	Tyrosine hydroxylase	
			Atrophic gastritis	H+/K+ pump ATPase	
			Pernicious anaemia	Intrinsic factor	
			Celiac disease	Transglutaminase	
			Autoimmune hepatitis	P450 (IID6, IA2)	
			Hypophysitis and so forth	68, 49, 43 kD from human	

**Table 3:** Impact of T1DM-associated autoimmune diseases on the course of T1DM and suggestion concerning screening tests.

T1DM-associated autoimmune diseases	Influence on the glucose metabolism	Impact on the course of T1DM	Suggestion of screening tests
Hashimoto thyroiditis hypothyroidism	↓intestinal glucose absorption ↓glycogenolysis ↓insulin catabolism ↑insulin sensitivity	Hypoglycemia	TSH, fT4, TPO Ab, and Tg Ab every year

Graves' disease hyperthyroidism	↓intestinal glucose absorption ↑insulin sensitivity and insulin metabolism in target tissue lipolysis	Hypoglycemia Ketoacidosis	TSH, fT4, fT3, and TSI by signs and symptoms thyrotoxicosis or by unexplained hypoglycemias

Celiac disease	↓intestinal glucose absorption	Hypoglycemia	Anti-transglutaminase antibodies TTG or IgA EMA over the years of the disease followed by biennial tests Gastroscopy with histopathological investigations of the intestinal mucosa by positive tests

Autoimmune gastritis	Pernicious anemia	—	—

Addison disease	↑insulin sensitivity ↓glycogenolysis ↓gluconeogenesis ↑glycolysis	Hypoglycemia	ACTH and cortisol levels by unexplained hypoglycemias or every 2-3 years

JIA, psoriasis, Sjogren syndrome, and other autoimmune diseases during corticosteroid therapy	↓insulin sensitivity ↑glycogenolysis ↑gluconeogenesis ↓glycolysis	Hyperglycemia	—
